# With the current COVID pandemic: should we use single-use flexible bronchoscopes instead of conventional bronchoscopes?

**DOI:** 10.1186/s13054-020-02965-9

**Published:** 2020-05-18

**Authors:** Patrick M. Honore, Aude Mugisha, Luc Kugener, Sebastien Redant, Rachid Attou, Andrea Gallerani, David De Bels

**Affiliations:** ICU Department, Centre Hospitalier Universitaire Brugmann-Brugmann University Hospital, Place Van Gehuchtenplein, 4, 1020 Brussels, Belgium

We would like to describe the numerous advantages of single-use bronchoscopes over conventional bronchoscopes especially during the COVID pandemic. Recently, Zaidi et al. did a comparative study between single-use and conventional bronchoscopes for bronchoalveolar lavage (BAL) [1]. They concluded that with single-use bronchoscopes, they achieved a larger BAL volume yield than conventional bronchoscopes, with comparable cell yield and viability [1]. Better volume yields may potentially reduce post-procedure side effects such as pleuritic chest pain and cough. With single-use devices, the risk of cross infection is eliminated, providing reassurance to researchers and participants [2]. This single-use flexible bronchoscope can be reusable for the same patient and should be stored in his isolate room [2]. Reduced maintenance requirements can be cost effective [3]. In addition, single-use flexible bronchoscopes have been evaluated in the critical care setting with favorable results for BAL, percutaneous tracheostomy, intubation, and suction [4]. Regarding the important question of cost, a recent study suggests benefits from the use of single-use flexible bronchoscopes in terms of cost effectiveness, cross-contamination, and resource utilization [3]. Single-use flexible bronchoscopes could be very useful in the setting of the current coronavirus pandemic. We have already started using them.

## Data Availability

Not applicable.

## Abbreviations

BAL: Bronchoalveolar lavage
ICU: Intensive care unit
